# Glucagon-like peptide-1 receptor agonist prevents pulmonary fibrosis following acute COVID-19 infection associated with type 2 diabetes

**DOI:** 10.1128/jvi.00401-26

**Published:** 2026-07-09

**Authors:** Runhong Zhou, Ming Yue, Qing Shen, Na Liu, Yuting Chen, Pui Wang, Kyungmin Kim, Ranyao Yang, Honglin Chen, Kwok-Yung Yuen, Kelvin Kai-Wang To, Aimin Xu, Zhiwei Chen

**Affiliations:** 1 AIDS Institute, School of Clinical Medicine, Li Ka Shing Faculty of Medicine, The University of Hong Kong 25809, Hong Kong Special Administrative Region, People’s Republic of China; 2 Department of Microbiology, School of Clinical Medicine, Li Ka Shing Faculty of Medicine, The University of Hong Kong 25809, Hong Kong Special Administrative Region, People’s Republic of China; 3 Centre for Virology, Vaccinology and Therapeutics, Hong Kong Science and Technology Park, Hong Kong Special Administrative Region, People’s Republic of China; 4 Department of Infectious Disease and Microbiology, The University of Hong Kong-Shenzhen Hospital 444333, Shenzhen, Guangdong Province, People’s Republic of China; 5 State Key Laboratory of Pharmaceutical Biotechnology, The University of Hong Kong 25809, Hong Kong Special Administrative Region, People’s Republic of China; 6 Department of Medicine, Li Ka Shing Faculty of Medicine, The University of Hong Kong 25809, Hong Kong Special Administrative Region, People’s Republic of China; 7 State Key Laboratory of Emerging Infectious Diseases, The University of Hong Kong 25809, Hong Kong Special Administrative Region, People’s Republic of China; 8 Pandemic Research Alliance Unit at The University of Hong Kong, Hong Kong Special Administrative Region, People’s Republic of China; Loyola University Chicago - Health Sciences Campus, Maywood, Illinois, USA

**Keywords:** SARS-CoV-2, pulmonary fibrosis, glucagon-like peptide-1 receptor agonist, post-acute sequelae of COVID-19, type 2 diabetes, proinflammatory macrophages

## Abstract

Post-acute sequelae of COVID-19 (PASC) poses a major health burden after SARS-CoV-2 infection. Although type 2 diabetes (T2D) is associated with PASC, the mechanism of T2D-mediated PASC in the lung remains elusive. Here, we found that people with T2D (PWT2D) exhibited significantly upregulated fibrosis-related genes in monocytes, which positively correlated with pulmonary fibrosis-related biomarkers up to 3 months after acute SARS-CoV-2 infection. Using db/db mice to model human T2D, we found consistently that SARS-CoV-2 infection resulted in upregulation of fibrosis-related genes in lung macrophages and persistent pulmonary fibrosis. Moreover, the macrophage-depletion demonstrated that pro-inflammatory macrophages in db/db mice were determinants for inducing pulmonary fibrosis post-infection. Importantly, the anti-T2D glucagon-like peptide-1 receptor agonist (GLP1-RA) reprogramed macrophage responses to SARS-CoV-2 by normalizing fibrosis-related genes, significantly reducing the pulmonary fibrosis in a glucose-independent manner. These findings demonstrated that SARS-CoV-2-induced proinflammatory macrophages are detrimental factors in T2D-mediated PASC, which can be prevented by GLP1-RA.

**IMPORTANCE:**

Some COVID-19 patients develop pulmonary post-acute sequelae of COVID-19 (PASC) with clinical symptoms lasting for years. Critically, the incidence of pulmonary PASC in PWT2D is four times higher than that in those without T2D. However, the immune mechanisms underlying pulmonary PASC in PWT2D remain poorly understood. Our findings demonstrate that SARS-CoV-2-induced proinflammatory macrophages are key drivers of PASC-associated pulmonary fibrosis. We further provide *in vivo* evidence that glucagon-like peptide-1 receptor agonists (GLP1-RAs) can reprogram pulmonary macrophages to prevent SARS-CoV-2-induced PASC in a T2D mouse model, with important implications for therapy in PWT2D.

## INTRODUCTION

Type 2 diabetes (T2D) is an escalating global health concern, affecting more than 6% of the world’s population (1). Prior to the COVID-19 pandemic, hyperglycemia in people with T2D (PWT2D) contributed to immune response dysfunction, resulting in infections that occurred more frequently and lasted longer than usual (2). Since the pandemic, COVID-19 patients with pre-existing T2D have shown worse clinical outcomes (3, 4). Infected PWT2D with poor glycemic control showed a significantly higher death rate (11.0% vs 1.1%) compared to those with good glycemic control (3). Although lymphocytopenia and a lack of cytotoxic CD8^+^ lymphocytes were associated with poor outcomes in both non-diabetic and T2D patients, larger monocyte size and the loss of peripheral classical monocytes (CD14^hi^ CD16^−^) were commonly observed in PWT2D under intensive care (5). The loss of peripheral classical monocytes is likely due to their differentiation into tissue macrophages mediated by CCL2 chemotaxis (6). Enhanced monocyte infiltration has been observed in multiple tissues of deceased COVID-19 patients, including the lungs, kidneys, and hearts (7).

Pulmonary fibrosis is one of the major post-acute sequelae of SARS-CoV-2 infection (PASC), accounting for 10% of infected people, which may persist for months or years as a condition termed “long COVID” (8, 9). PASC affects the respiratory, cardiovascular, neurological, psychiatric, digestive, and other systems (8–10). While T2D might enhance fibrosis in various systems (11), persistent lung fibrosis was the major sequelae of acute respiratory distress syndrome (12–14). As the primary organ attacked by SARS-CoV-2, the lung displayed damage in parenchyma and interstitial tissues, resulting in pulmonary fibrosis with prolonged symptoms such as coughing, shortness of breath, chest pain, excessive sputum, and chest tightness (10, 15–18). Persistent lung diffusion impairment with evolving fibrosis was observed in individuals even 12 months post-infection (19, 20). Several studies reported a high incidence of PASC in PWT2D (4, 21). D-dimer, a biomarker of pulmonary fibrosis, is significantly higher levels in PWT2D during acute infections (3). Poorly controlled T2D was associated with a higher incidence of pulmonary fibrosis in PWT2D, who experienced persistent breathlessness and required oxygen supplementation during the post-COVID-19 period (21–24). Although hyperglycemia in PWT2D is known to augment monocyte pro-inflammatory cytokine responses (25), the mechanism underlying T2D-mediated persistent pulmonary fibrosis remains incompletely investigated.

Glucagon-like peptide-1 receptor agonists (GLP1-RAs) provide not only glycemic control and weight reduction but also cardiovascular and renal protection (26). Beyond these metabolic benefits, emerging evidence supports their anti-inflammatory and immunomodulatory effects (27). Recent studies have shown that GLP1 receptor activation can attenuate systemic inflammation and pulmonary fibrosis through mechanisms involving central nervous system–mediated adrenergic and opioid GPCR signaling, as well as inhibition of NLRP3 inflammasome activation (28, 29). These findings highlight the potential therapeutic utility of GLP1-RAs in mitigating SARS-CoV-2–induced lung injury.

An analysis of retrospective cohorts and bleomycin-induced pulmonary fibrosis in a murine model, both innate and adaptive immune systems, has been implicated in the pathogenesis of pulmonary fibrosis. Increased counts of monocytes and inflammatory macrophages, as well as the involvement of T cells and IgA, have been associated with the risk of exacerbations and progression of pulmonary fibrosis (24, 30–33). In this study, we investigated the role of T2D in PASC-associated pulmonary fibrosis. We demonstrated that proinflammatory macrophages in PWT2D are determinants of PASC-associated pulmonary fibrosis. We also discovered that a GLP1-RA reprograms macrophage responses to SARS-CoV-2 by normalizing fibrosis-related genes, thereby significantly reducing pulmonary fibrosis.

## RESULTS

### PWT2D with PASC show high expression of fibrosis-related genes in monocytes

**Fig 1:**
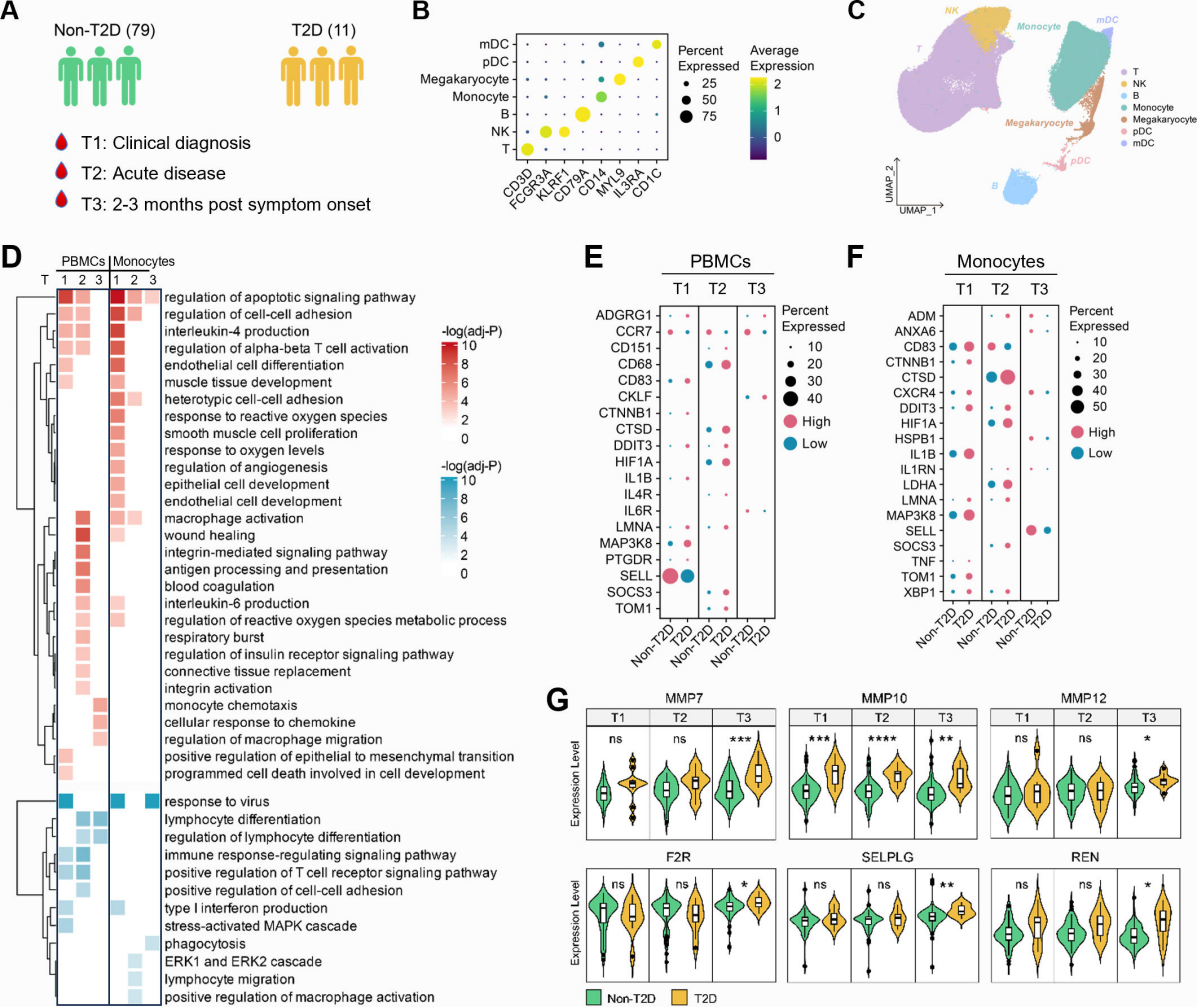
PWT2D with PASC show high expression of fibrosis-related genes in monocytes. (**A**) Overview of the cohort subjects (non-T2D, *n* = 79; T2D: *n* = 11) for single-cell RNA sequencing reanalysis (Accession: E-MTAB-10129). Samples were collected at three timepoints: T1 (baseline at clinical diagnosis/hospital admission), T2 (acute disease, around 5 days post-admission), and T3 (convalescent stage, 2–3 months post-symptom onset). (**B**) Dot plot of representative markers for each cell type. The size of dots represents the percentage of cell expression. (**C**) UMAP visualization of seven identified immune cell groups pooled from PBMCs of non-T2D/T2D donors. (**D**) Heatmaps of the enriched GO pathways in the comparison between non-T2D and T2D across the timepoints. The red squares represent upregulated pathways in the T2D group, while the blue ones represent downregulation. (**E and F**) Dot plots of differentially expressed fibrosis-related genes across donors and timepoints in PBMCs (**E**) and monocytes (**F**). Statistically significance was determined by the Wilcoxon rank-sum test with Bonferroni-adjusted *P*-value < 0.05. The size of dots represents the percentage of cell expression. The red dots represent relatively higher expression levels in the comparisons. (**G**) Violin plots of the fibrosis-related plasma biomarkers across donors and timepoints. *P*-values calculated from the t-test are displayed. ns *P* > 0.05, *P < 0.05, **P < 0.01, ***P < 0.001, ****P < 0.0001.

A significantly higher prevalence of persistent respiratory symptoms has been documented among PWT2D with PASC than that in non-T2D people (34, 35). To investigate the mechanisms underlying T2D-mediated PASC, we performed a reanalysis of a previously published longitudinal single-cell RNA sequencing data set (Accession: E-MTAB-10129) (34). This data set profiled peripheral blood mononuclear cells (PBMCs) from COVID-19 patients across three distinct time points: T1 (baseline at clinical diagnosis/hospital admission), T2 (acute disease, around 5 days post-admission), and T3 (convalescence, 2–3 months post-symptom onset) originally. From this cohort, we specifically selected samples with available diabetes annotations for downstream analysis. This selection resulted in a focused cohort of non-T2D subjects (*n* = 79) and PWT2D (*n* = 11) (Fig. 1A). UMAP visualization revealed seven distinct clusters of immune cells including T cells (CD3D), NK cells (FCGR3A and KLRF1), B cells (CD79A), monocytes (CD14 high), megakaryocytes (CD14 dim and MYL9), plasmacytoid dendritic cells (pDC) (IL3RA), and myeloid dendritic cells (mDC) (CD14 dim and CD1C) (Fig. 1B and C). However, an insignificant increase was observed only in monocytes from patients with T2D compared to non-T2D patients at T2 and T3 (Fig. S1A). We then compared the GO pathways between non-T2D and PWT2D at these three timepoints (Fig. 1D). Consistent with previous findings that PWT2D had poor immune responses after viral infection (36), a significant downregulation of innate and adaptive immune response-related GO pathways (blue dots) (e.g., Type I interferon production and T-cell receptor signaling) was observed in PWT2D with PASC at all three timepoints. The GO pathways required for respiratory tissue repair, including cell-cell adhesion, endothelial cell differentiation, muscle tissue development, regulation of epithelial to mesenchymal transition, wound healing, and integrin activation, were significantly upregulated in PWT2D with PASC from T1 to T2 but returned to a steady level at T3 (red dots). In contrast, the GO pathways related to monocyte chemotaxis, cellular responses to chemokines, and macrophage migration were upregulated in PWT2D with PASC at T3, indicating the possible differentiation of monocytes to macrophages and migration to effector sites (e.g., lung). These findings explain the increase in monocyte frequency at T2 and T3 (Fig. S1A). Plasma proteomics analysis also revealed the macrophage colony-stimulating factor, CSF1, which was highly expressed in PWT2D at T1 and T2 (Fig. S1B). CSF1 is a master regulator of macrophage differentiation (37). Plasma inflammatory proteins expressed at T1 and T2 may return afterward to similar amounts as in healthy donors despite the presence of ongoing respiratory morbidity (38). We also observed higher levels of chemokines (CCL11, CCL2, and CXCL9) and proinflammatory cytokines (IL-1, IL-6, IL-17, IL-18, TGF-A, and TNF) in PWT2D at T1 and T2 (Fig. S1B). Given that the monocyte infiltration is associated with respiratory distress (39, 40), analysis of GO pathways in monocytes showed weak innate immune responses, such as lower type I interferon production in PWT2D with PASC. However, they show stronger production of IL-6 and reactive oxygen species (ROS) at T1, which are key mediators of inflammation-associated tissue damage (41, 42). Consistently, fibrosis-related genes, including cathepsin D (CTSD), HIF-1α, and lactate dehydrogenase A (LDHA), were highly expressed in monocytes derived from PWT2D with PASC compared to non-T2D with PASC at T2 (Fig. 1E and F). In addition, most pulmonary fibrosis-associated biomarkers, including MMPs, F2R, SELPLG, and REN, were higher in PWT2D than in non-T2D patients at T3 (Fig. 1G). These results indicated that monocytes in PWT2D are actively involved in the respiratory tissue damage and repair processes associated with PASC pulmonary fibrosis after SARS-CoV-2 infection.

### SARS-CoV-2-infected T2D mice exhibit increased pulmonary fibrosis-related fibronectin gene

**Fig 2:**
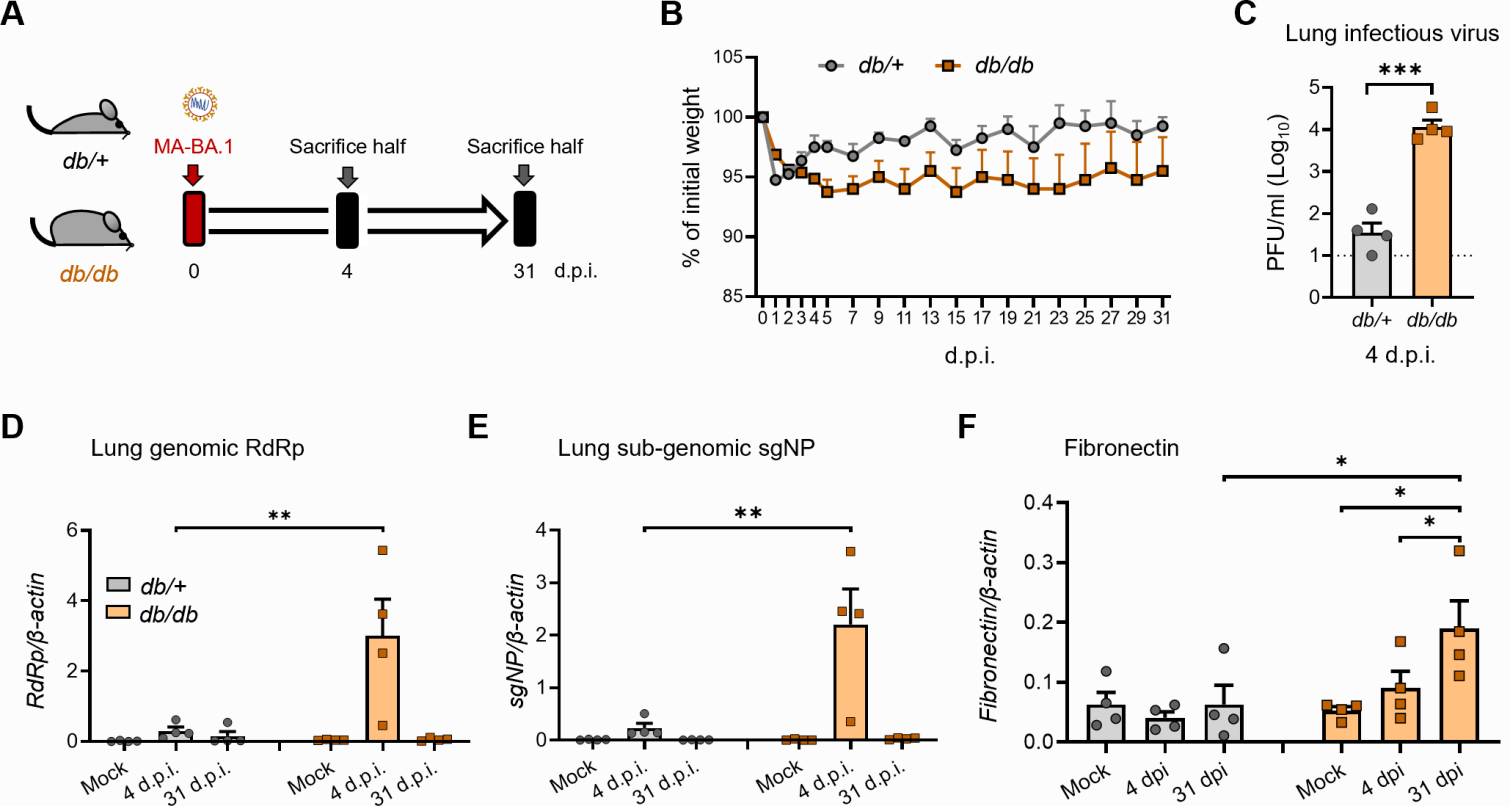
SARS-CoV-2-infected T2D mice exhibit increased pulmonary fibrosis-related fibronectin gene. (**A**) Schedule of the T2D mouse study. db/+ and db/db mice (*n* = 8) were intranasally infected with 10^5^ PFU of mouse-adapted Omicron BA.1 (MA-BA.1). Half the number of mice were sacrificed at 4 and 31 d.p.i. for analysis, respectively. (**B**) Body weight was measured according to day post-infection (d.p.i.). (**C**) Infectious viruses were quantified in lung homogenates by the viral plaque assay. Dash line indicates the limit of detection. (**D and E**) Sensitive RT-PCR was used to quantify the copy numbers (normalized by β-actin) of SARS-CoV-2 genomic RdRp RNA (**D**) and NP subgenomic RNA (**E**), as well as of fibronectin gene (**F**) in lung homogenates at 4 and 31 d.p.i. Mock groups of uninfected mice were included as controls. Statistics were generated using t-test. ∗*P* < 0.05, ∗∗*P* < 0.01, ∗∗∗*P* < 0.001.

To model the pulmonary fibrosis found in PWT2D after SARS-CoV-2 infection, we tested db/db mice, which is a well-established animal model for studying phases 1 to 3 T2D due to a spontaneous mutation in the leptin receptor (43). db/db mice and the non-diabetic control mice (db/+) were intranasally infected with 10^5^ PFU of mouse-adapted SARS-CoV-2 Omicron BA.1 (MA-BA.1) (44). Half of the infected animals were sacrificed for analysis at 4 (acute) and 31 (long-term) days post-infection (d.p.i.) (Fig. 2A). Infection caused a greater reduction in body weight in db/db mice than in db/+ mice. Of note, the body weight of db/+ mice reached 100% of the initial value at 13 d.p.i., whereas long-lasting weight loss (~5%) was observed in all db/db mice up to 31 d.p.i., suggesting that db/db mice had more severe and persistent disease than db/+ mice after SARS-CoV-2 infection (Fig. 2B). A significantly higher live virus titer was observed in the lungs of db/db mice than in the lungs of db/+ mice at 4 d.p.i. (Fig. 2C). Significantly higher viral RNA copies (genomic RdRp and sub-genomic sgNP) were consistently found in db/db mice at 4 d.p.i. than in db/+ mice (Fig. 2D and E). Viral RNA in the lungs was undetectable at 31 d.p.i. in all the mice. Similar to the findings in PWT2D, db/db mice had lower levels of neutralizing antibodies than those in db/+ mice (Fig. S2A). By analyzing the RNA profile of the lungs, we further found that the fibrosis-related fibronectin gene was only enhanced in db/db but not in db/+ mice in a time-dependent manner up to 31 d.p.i. (Fig. 2F). These findings demonstrated that the T2D-mediated PASC pulmonary fibronectin gene was increased in SARS-CoV-2-infected db/db mice.

### Long-lasting lung injury with fibrosis in T2D mice after SARS-CoV-2 infection

**Fig 3:**
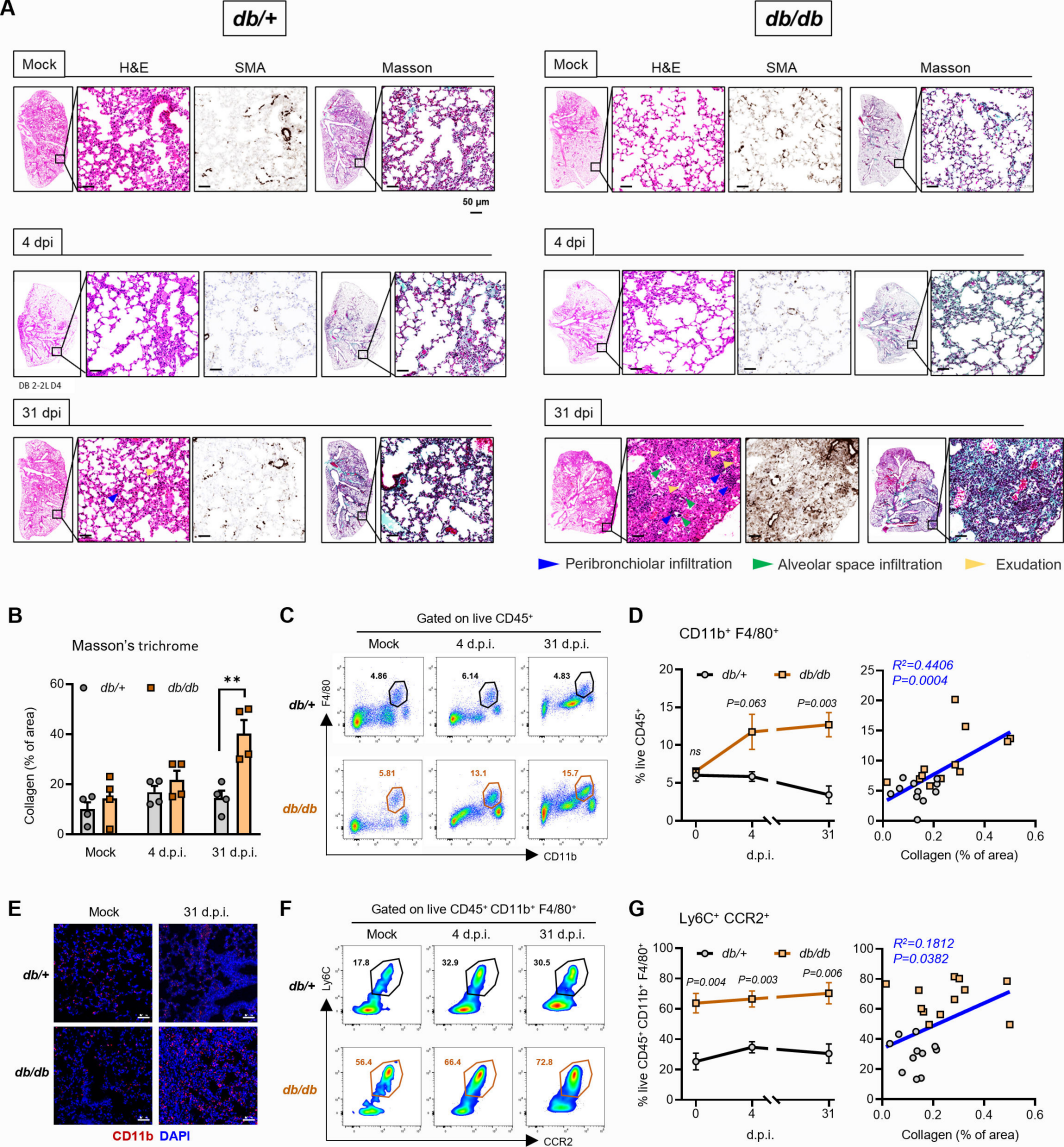
Long-lasting lung injury with fibrosis in T2D mice after SARS-CoV-2 infection. (**A**) Histopathological analysis of lungs at the indicated timepoints. H&E means hematoxylin and eosin staining. α-SMA (SMA) staining was visualized from fluorescence data using Phenochart Simulated Brightfield mode, rendering SMA-positive cells in a brown IHC-like signal. Masson’s trichrome staining shows the collagen. Scale bars, 50 µm. (**B**) Percentages of the collagen area relative to the lung area were quantified by ImageJ. (**C**) Representative plots show the frequencies of CD11b^+^F4/80^+^ macrophages in lungs. (**D**) Frequencies of CD11b^+^F4/80^+^ macrophages were gated on CD45^+^ live cells in db/+ and db/db mice at 0, 4, and 31 d.p.i., and their correlation with collagen in lung macrophages. (**E**) Confocal images show CD11b^+^ cells (red) in lungs. Scale bars: 50 µm. (**F**) Representative plots show the frequencies of Ly6C- and CCR2-expressing cells. (**G**) Frequencies of Ly6C^+^CCR2^+^ MDM and their correlation with collagen are shown in CD45^+^ live cells of db/+ and db/db mice at 0, 4, and 31 d.p.i. Statistics were generated using a t-test. ∗*P* < 0.05, ∗∗*P* < 0.01.

To understand tissue injury in SARS-CoV-2-infected db/db mice, we performed a pathological analysis. We found that db/db mice displayed long-lasting lung injury with peribronchiolar infiltration, alveolar space infiltration, and exudation at 31 d.p.i. (Fig. 3A). In contrast, only mild peribronchiolar infiltration was observed in db/+ mice. Importantly, α-smooth muscle actin (α-SMA), a marker of activated fibroblasts, increased extensively in the lungs of db/db mice but not in those of db/+ mice at 4 and 31 d.p.i. (Fig. S2B). Masson’s trichrome staining confirmed that db/db mice had a significantly higher percentage of collagen area in lungs compared to db/+ mice at 31 d.p.i. (Fig. 3B). These results demonstrated that db/db mice developed severe pulmonary fibrosis after the acute phase of SARS-CoV-2 infection.

To understand the cellular mechanism underlying the T2D-mediated long-lasting pulmonary fibrosis, we further analyzed the profiles of immune cells in the lungs of both db/+ and db/db mice. No significant differences were observed in most types of immune cells between db/+ and db/db mice at 4 and 31 d.p.i. (Fig. S3). The frequency of CD11b^+^F4/80^+^ macrophages, however, was significantly increased at 4 d.p.i. and remained increased to 31 d.p.i. in db/db mice compared with db/+ mice (Fig. 3C and D). This finding was confirmed by confocal imaging of staining CD11b in the lung tissues (Fig. 3E). Notably, the majority of these infiltrated lung macrophages (>60%) in db/db mice showed the Ly6C^+^CCR2^+^ monocyte-derived macrophage (MDM) phenotype before SARS-CoV-2 infection (0 d.p.i.). After infection, the mean frequency of Ly6C^+^CCR2^+^ lung MDMs in db/db mice continued to increase and was significantly higher than that in db/+ mice at 31 d.p.i. (Fig. 3F and G). Correlation analysis then demonstrated that both the frequency of CD11b^+^F4/80^+^ macrophages (Fig. 3D, right) and the frequency of Ly6C^+^CCR2^+^ macrophages (Fig. 3G, right) were positively correlated with the percent collagen area in the lungs. These results demonstrated that MDMs enhanced by SARS-CoV-2 infection contribute significantly to T2D-mediated long-lasting pulmonary fibrosis.

### Macrophage depletion prevented pulmonary fibrosis in db/db mice after SARS-CoV-2 infection

**Fig 4:**
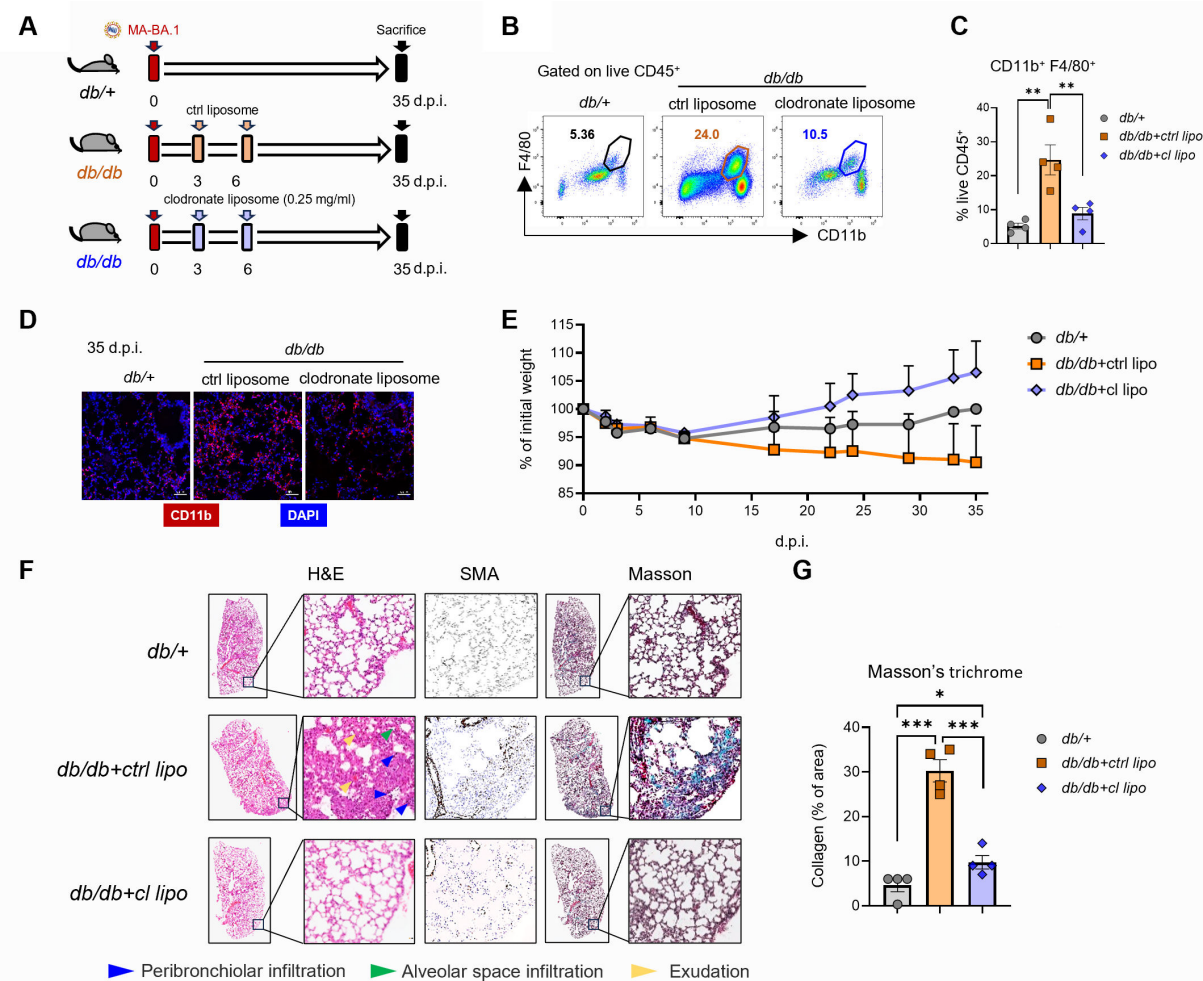
Macrophage depletion prevented pulmonary fibrosis in db/db mice after SARS-CoV-2 infection. (**A**) Schedule of the mouse study. db/+ and db/db mice were infected with 10^5^ PFU of MA-BA.1. db/db mice were then intranasally inoculated with 50 µL of control liposome (ctrl lipo) or clodronate liposome (cl lipo) (0.25 mg/mL) at 3 and 6 d.p.i. Mice were sacrificed at 35 d.p.i. for analysis. (**B**) Representative plots show the frequencies of CD11b^+^F4/80^+^ macrophages in the lung. (**C**) Frequencies of CD11b^+^F4/80^+^ macrophages are shown in CD45^+^ live cells derived from db/+ and db/db mice at 0, 4, and 31 d.p.i. (**D**) Confocal images show CD11b^+^-positive (red) cells in lungs. Scale bars: 50 µm. (**E**) Body weight changes are measured after viral infection. (**F**) Histopathological analysis of lungs is shown at the indicated timepoints. H&E means hematoxylin and eosin staining. α-SMA (SMA) staining was visualized from fluorescence data using Phenochart Simulated Brightfield mode, rendering SMA-positive cells in a brown IHC-like signal. Masson’s trichrome staining depicts the collagen. Scale bars, 50 µm. (**G**) Percentages of the collagen area relative to the lung area are quantified by ImageJ. Statistics were generated using a t-test. ∗*P* < 0.05, ∗∗*P* < 0.01.

To determine the contribution of macrophages in SARS-CoV-2-induced pulmonary fibrosis, we performed *in vivo* depletion of pulmonary phagocytic cells, predominantly macrophages, by intranasal administration of clodronate liposomes (cl lipo, 0.25 mg/mL) or control liposomes (ctrl lipo) twice, with a 3-day interval, beginning at 3 d.p.i. after SARS-CoV-2 infection (Fig. 4A) (45). We first confirmed that cl lipo administration significantly reduced the frequency of macrophages in the lungs of db/db mice at 35 d.p.i. by flow cytometry (Fig. 4B and C) and confocal imaging (Fig. 4D), respectively. The db/db mice treated with ctrl lipo showed persistent weight loss (<90.5% of the initial weight) compared to db/+ mice. The cl lipo treatment improved the weight recovery of db/db mice up to 106.5% of the initial weight (Fig. 4E). Pathological analysis then revealed that depleting macrophages by cl lipo significantly reduced macrophage peribronchiolar infiltration, alveolar space infiltration, and exudation in db/db mice with less production of α-SMA (Fig. 4F). Moreover, cl lipo significantly reduced collagen levels in the lungs of db/db mice (Fig. 4G). These results demonstrated that macrophages recruited by SARS-CoV-2 infection acted as a detrimental contributor to T2D-associated long-lasting pulmonary fibrosis.

### GLP1-RA prevented pulmonary fibrosis in db/db mice after SARS-CoV-2 infection

**Fig 5:**
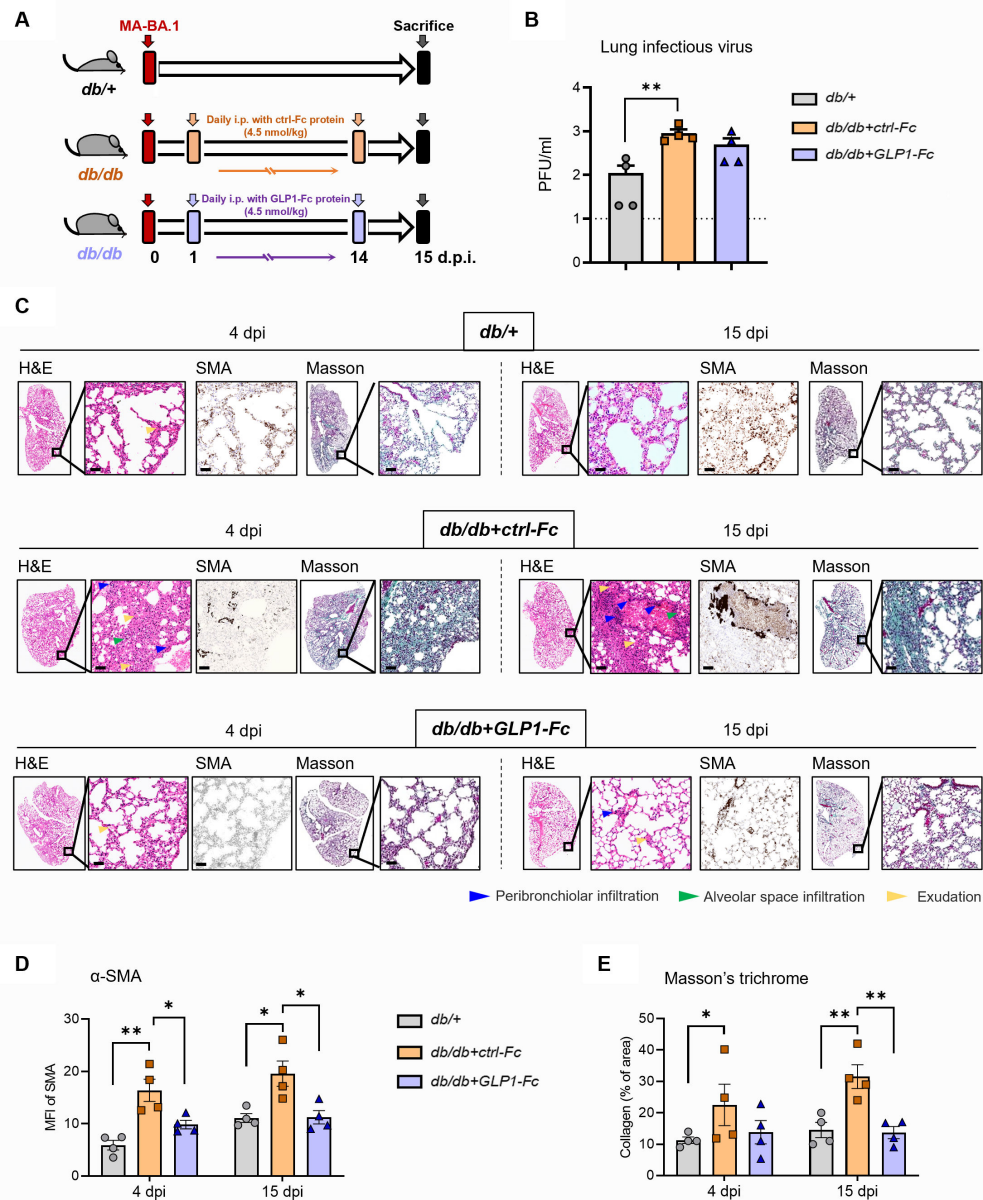
GLP1-RA prevented pulmonary fibrosis in db/db mice after SARS-CoV-2 infection. (**A**) Schedule of the mouse study. db/+ and db/db mice were intranasally infected with 10^5^ PFU Omicron BA.1. db/db mice received daily i.p. injection with 45 nmol/kg of ctrl-Fc or GLP1-Fc from 1 d.p.i. Half of the mice were sacrificed at 4 and 15 d.p.i. for analysis. (**B**) Infectious viruses were quantified in lung homogenates by the viral plaque assay. (**C**) Histopathological analysis of lungs is shown for the indicated timepoints. H&E means hematoxylin and eosin staining. α-SMA (SMA) staining was visualized from fluorescence data using Phenochart Simulated Brightfield mode, rendering SMA-positive cells in a brown IHC-like signal. Masson’s trichrome staining indicated the collagen. Scale bars, 50 µm. (**D**) The extent of α-SMA staining in the lungs was quantified as MFI in db/+ and db/db mice. (**E**) Percentages of the collagen area relative to the lung area were quantified by ImageJ. Statistics were generated using a t-test. ∗*P* < 0.05, ∗∗*P* < 0.01.

GLP1-RA has been used to treat T2D because of its ability to reduce glucose and its anti-inflammatory effects (46). Since the GLP1 receptor is highly expressed in lung tissue (47), we sought to determine the role of GLP1-RA (e.g., GLP1-Fc) in preventing SARS-CoV-2-induced pulmonary fibrosis in db/db mice. Animals were intraperitoneally injected with ctrl-Fc or GLP1-Fc daily from 1 to 14 d.p.i. The mice were then sacrificed at 4 and 15 d.p.i. for analysis (Fig. 5A). GLP1-Fc treatment exhibited a minor reduction in virus titers in the lungs of db/db mice compared with ctrl-Fc treatment at 4 d.p.i. (mean 900 vs 500 PFU/mL) (Fig. 5B). We then measured the non-fasting blood glucose levels at the acute phase after infection (4 d.p.i.) and found that GLP1-Fc treatment decreased the non-fasting blood glucose levels without significance compared to ctrl-Fc treatment (Fig. S4A), although the non-fasting blood glucose level was positively correlated with lung viral titer (Fig. S4B). Moreover, GLP1-Fc treatment reduced diffuse alveolar damage, decreased immune cell accumulation, and reduced the production of α-SMA, thereby preventing collagen deposition in the lungs of db/db mice at 15 d.p.i. compared to that in the ctrl-Fc group (Fig. 5C through E). Although confocal imaging revealed a comparable number of CD11b^+^ cells in the lungs of mice that received either ctrl-Fc or GLP1-Fc during the acute phase (4 d.p.i.), a higher infiltration of CD11b^+^ cells including MDMs was exclusively observed in the lungs of ctrl-Fc mice than in GLP1-Fc mice during the recovery phase (15 d.p.i.) (Fig. S5). These results demonstrated that GLP1-RA can prevent pulmonary fibrosis in db/db mice after SARS-CoV-2 infection.

### GLP1-RA reprogramed macrophage response to SARS-CoV-2

**Fig 6:**
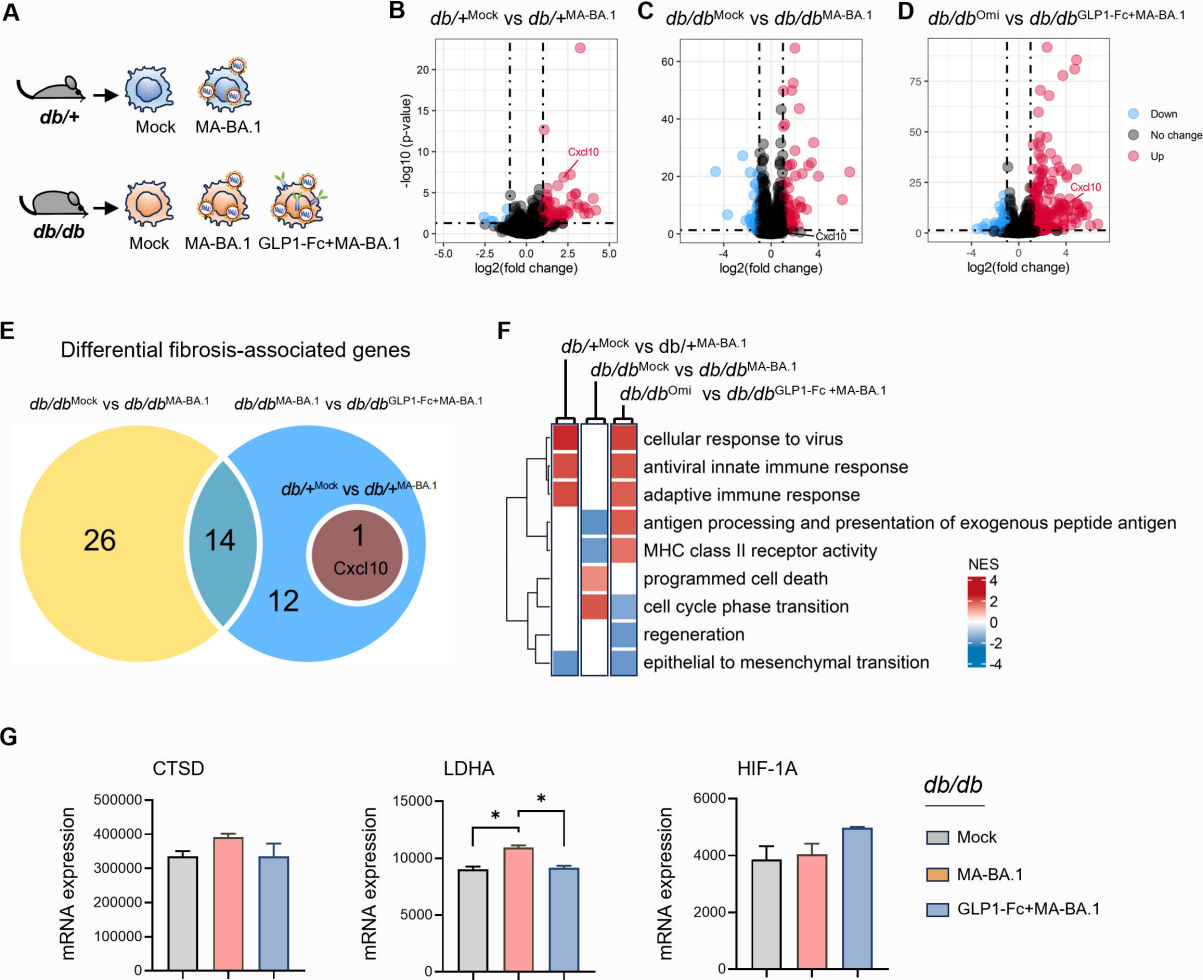
GLP1-RA reprogramed macrophage response to SARS-CoV-2. (**A**) Experimental design of RNA sequencing of macrophages. BMDM differentiated from db/+ and db/db mice were infected with Mock or MA-BA.1 in the presence of ctrl-Fc or GLP1-Fc, respectively. RNA samples were isolated at 1 d.p.i. (**B–D**) Volcano plots reflect the changes in Cxcl10 expression in experimental groups. (**E**) A Venn diagram shows differential fibrosis-associated genes compared between experimental groups. (**F**) Heatmap illustrates the GSEA NES results. Red and blue intensities represent downregulation and upregulation of different pathways, respectively. (**G**) The mRNA expression of fibrosis genes in db/db BMDMs after MA-BA.1 infection in the presence of ctrl-Fc or GLP1-Fc, respectively. Statistics were generated using a t-test. ∗*P* < 0.05 and ∗∗∗*P* < 0.001.

Since GLP1-RA is unlikely to directly deplete the lung macrophages, we sought to explore its mode of action *in vivo*. We observed that the granulocyte-monocyte progenitors (GMPs) cells, the progenitors of monocytes that further differentiate into macrophages and dendritic cells, were significantly increased in the bone marrow after SARS-CoV-2 infection (Fig. S6A and B). We hypothesized that bone marrow GMPs might be a source of MDMs that infiltrate the lungs after SARS-CoV-2 infection. We, therefore, exposed bone marrow-derived macrophages (BMDM) isolated from db/+ and db/db mice with SARS-CoV-2 (MOI: 1) in the presence of ctrl-Fc or GLP1-Fc (Fig. 6A). Macrophages from db/db mice exhibit persistent epigenetic changes in innate immunity (48). We hence analyzed the specific and common gene changes between our experimental groups by bulk RNA sequencing. We found that BMDM derived from db/+ mice after infection (db/+^MA-BA.1^) showed significantly increased CXCL10 expression compared to those from db/+ mice without infection (db/+^Mock^) (Fig. 6B). However, CXCL10 expression was not upregulated in BMDM derived from db/db mice after infection (Fig. 6C). In contrast, GLP1-Fc treatment resulted in high CXCL10 expression in BMDM derived from db/db mice after infection (Fig. 6D). These results demonstrated that GLP1-Fc may prevent SARS-CoV-2-induced pulmonary fibrosis in db/db mice by reprogramming BMDM for CXCL10 induction to inhibit pulmonary fibrosis (49).

Further intergroup comparisons revealed that 26 fibrosis-related genes were significantly reprogrammed in db/db-derived BMDM after SARS-CoV-2 infection (Fig. 6E, yellow pie). Although 14 fibrosis-related genes were maintained at a similar stage in both the db/db^Mock^ vs db/db^ctrl-Fc+MA-BA.1^ BMDM and db/db^ctrl-Fc+MA-BA.1^ vs db/db^GLP1-Fc+MA-BA.1^ BMDM (dark blue pie), 12 fibrosis-related genes were identified in db/db^ctrl-Fc+MA-BA.1^ vs db/db^GLP1-Fc+MA-BA.1^ BMDM (bright blue pie) including CXCL10 (brown pie), indicating that GLP1-Fc treatment changed the infection-induced fibrosis-related genes (Fig. 6E). The heatmap generated by the Gene Set Enrichment Analysis (GSEA) indicated that SARS-CoV-2 infection readily induced antiviral responses, including cellular responses to virus, antiviral innate immune response, and adaptive immune response in db/+-derived BMDM (Fig. 6F, left column). Such antiviral responses were not observed in db/db-derived BMDM treated with ctrl-Fc (Fig. 6F, middle column). In contrast, db/db-derived BMDMs treated with GLP1-Fc were reprogrammed to generate antiviral responses while downregulating fibrosis-related gene sets (Fig. S7), including cell cycle phase transition, regeneration, and epithelial to mesenchymal transition (Fig. 6F, right column). Consistently, GLP1-Fc treatment suppressed the fibrosis genes CTSD and LDHA in db/db mouse BMDM after SARS-CoV-2 infection, instead of HIF1A (Fig. 6G). These fibrosis genes were also found in monocytes derived from the T2D PASC cohorts (Fig. 1F). These results demonstrated that GLP1-RA reprogramed db/db-derived BMDM by increasing antiviral responses while suppressing some fibrosis-related genes.

## DISCUSSION

Millions of COVID-19 survivors are suffering from PASC that persists for months to years following SARS-CoV-2 infection (50). Although 90% of people with PASC experienced mild COVID-19, hospitalized patients had a significantly higher incidence of PASC for years, especially concerning pulmonary fibrosis (51–53). To this end, PWT2D showed worse clinical outcomes and mortality (3, 4). Moreover, pulmonary fibrosis in PWT2D was associated with highly upregulated plasma biomarkers involved in epithelial damage and repair (e.g., MMPs) in the late phase (2–3 months post-symptom onset) (38). Cough is one of the most common symptoms of pulmonary fibrosis (54). Studies on PWT2D have revealed a significant association between T2D and PASC with fatigue, cough, and respiratory viral symptoms (21–23, 34). Although several risk factors (e.g., aging, gender, immunodeficiency, autoimmunity, T2D) were associated with PASC (4, 8, 34, 35, 50, 53, 55–58), we consistently report that PWT2D with PASC expressed significantly higher amounts of fibrosis-related genes in PBMCs and monocytes compared to non-T2D individuals in the acute disease phase. Critically, fibrosis-related genes in monocytes were positively correlated with pulmonary fibrosis biomarkers in infected PWT2D. These findings suggested that preventing pulmonary fibrosis in PWT2D with COVID-19 becomes a practical strategy for the fight against T2D-mediated PASC.

To investigate the mechanism of T2D-mediated PASC, we successfully established a relevant murine T2D model using db/db mice infected with a mouse-adapted SARS-CoV-2 Omicron strain (MA-BA.1). We focused on PASC in db/db mice with COVID-19 characterized by pulmonary fibrosis at 15–35 d.p.i. as described in previous animal studies (59–62). We demonstrated that SARS-CoV-2-infected db/db mice developed long-term weight loss and pulmonary fibrosis. These symptoms were similar to those of PASC found in PWT2D. These results are also consistent with those of a cohort study in which the Omicron variant may cause PASC in PWT2D, although it was suggested to have lower pathogenicity than the pre-Omicron viral strains (55).

Proinflammatory macrophages are detrimental factors to T2D-associated pulmonary fibrosis in infected db/db mice. Several animal studies have demonstrated acute lung injury in diabetic mice only up to 7 days after SARS-CoV-2 infection, but the underlying mechanism of T2D-associated PASC remains elusive (63–66). Several risk factors might contribute to pulmonary fibrosis in db/db mice, similar to those in patients with PASC. For example, viral load, which was detected at high levels in db/db mice, has been significantly associated with the development of PASC in patients (56). A low amount of neutralizing antibodies, found in our db/db mice, has been predictive of PASC at 6–7 months in both hospitalized and non-hospitalized patients (67). Poor cytotoxic T cells may also contribute to PASC (34, 68). In this study, we demonstrated that proinflammatory Ly6C^+^CCR2^+^ macrophages were predominantly recruited and maintained in the lungs of db/db mice from 4 d.p.i. to 35 d.p.i., which was consistent with the clinical findings of proinflammatory monocytes and macrophages infiltrated in the lungs of patients with pulmonary fibrosis and PASC (18, 38, 40, 69). We also demonstrated that these proinflammatory macrophages were responsible for SARS-CoV-2-induced pulmonary fibrosis, especially by the macrophage depletion experiment. To some extent, our findings are in line with recent reports that proinflammatory macrophages are the major causative factor of impaired skin healing in diabetes and obesity, as recently reported (69, 70). Notably, similar fibrosis gene signatures (CTSD and LDHA) observed in PASC patients were consistently found in macrophages from db/db mice infected with mouse-adapted SARS-CoV-2 (34). Taken together, our results not only revealed a mechanism by which proinflammatory macrophages are detrimental factors to T2D-mediated PASC in the lungs of infected db/db mice but also provided a useful *in vivo* model to search for biomedical interventions to prevent pulmonary PASC in PWT2D.

GLP1-RA prevented the T2D-associated pulmonary fibrosis and PASC. To date, there are no specific drugs or targeted therapies available for PASC prevention (71). Paxlovid has been shown to provide some protection against cognitive and fatigue but not the respiratory symptoms of PASC (72). Nintedanib, an FDA-approved antifibrotic agent, did not affect weight loss, recovery, or viral clearance, but it did reduce tissue congestion, fibrotic prevalence, and collagen deposition in aged mice (59). Recently, metformin, a first-line medication often prescribed for individuals with diabetes, was shown to reduce the incidence of PASC by approximately 41% when started within 3 days of symptom onset in a large cohort (*n* = 1,431) (73). The antifibrotic effects of metformin may be associated with reduced glucose absorption and aberrant extracellular matrix (ECM) remodeling in various tissues, including lung tissues, in different experimental models (74, 75). Metformin is known to increase GLP1 secretion (76). We speculated that metformin might act through increased secretion of GLP1, contributing to the antifibrotic effects in T2D. Moreover, GLP1-RAs have inhibitory effects on local or systemic inflammation by modulating different types of immune cells (29, 77, 78). We here tested the ability of GLP1-RA to prevent pulmonary fibrosis in db/db mice. We found that GLP1-RA treatment only partially decreased non-fasting blood glucose during acute infection, suggesting the glucose-independent effects of GLP1-RA in preventing pulmonary fibrosis in db/db mice. We then demonstrated that GLP1-RA reprogrammed BMDM by inducing CXCL10 to inhibit pulmonary fibrosis in infected db/db mice (49), highlighting an additional role in regulating macrophages. This extends previous work showing that GLP1 receptor activation attenuates the progression of pulmonary fibrosis via inhibition of inflammasome activation in lung fibroblasts (28).

Overall, our study demonstrated the essential role of proinflammatory macrophages in T2D-associated long-term lung injury after SARS-CoV-2 infection and discovered the therapeutic potential of GLP1-RA in preventing pulmonary fibrosis and PASC in PWT2D.

### Limitations

Due to the limited availability of mouse-adapted SARS-CoV-2 variants, we were unable to determine the impact of pre-Omicron and recent variants on PASC in db/db mice. The impact of GLP1-RA on other immune cells, including T cells, dendritic cells, and IgA-producing B cells associated with the progression of pulmonary fibrosis, requires future investigation (24, 30–33, 79). For example, we observed a significant upregulation of CD11b^+^ dendritic cells in db/db mice at 4 dpi, but their role in T2D-associated PASC remains unknown. In addition, although GLP1-RA prevented pulmonary collagen deposition at 15 dpi, longer-term studies are required to fully assess its sustained antifibrotic effects. Future studies will also be needed to test the efficacy of combining GLP1-RA with other anti-diabetic drugs (e.g., metformin and sodium-glucose co-transporter-2 inhibitors).

## MATERIALS AND METHODS

### Cell lines

HEK 293T-hACE2 and Vero-E6-TMPRSS2 cells (mycoplasma negative) were maintained in Dulbecco’s Modified Eagle Medium (DMEM) (Thermo Fisher Scientific) containing 10% fetal bovine serum, 2 mM L-glutamine, and 100 U/mL penicillin and were incubated at 37°C in 5% CO2 setting. High-level and stable expression of human ACE2 on HEK 293T-hACE2 cell line surface was validated by flow cytometry analysis.

### Live virus

The mouse-adapted SARS-CoV-2 Omicron BA.1 (MA-BA.1) was obtained from Dr. Pui Wang, who has been used previously (44). The sequence of the virus is available in GenBank (accession number OQ619133) and the GISAID database (accession number EPI_ISL_12996408). Confluent Vero-E6-TMPRSS2 cells were infected at 0.01 MOI with MA-BA.1. After 3 days of incubation, virus supernatant was collected for titration by plaque assay using Vero-E6-TMPRSS2 cells.

### Animal studies

All animal experimental protocols were approved by the Committee on the Use of Live Animals in Teaching and Research (CULATR 23-155) by the Centre for Comparative Medicine Research at the University of Hong Kong. For the SARS-CoV-2 challenge, 4-month-old female db/+ and db/db mice were anesthetized by a mixture of ketamine/xylazine solution (60 mg/kg and 10 mg/kg) and intranasally infected with MA-BA.1 at a dose of 1 × 10^5^ PFU. For the GLP1-RA treatment experiment, infected animals were intraperitoneally injected with ctrl-Fc or GLP1-Fc (4.5 nM/kg) daily from 1 to 14 d.p.i. For the macrophage depletion experiment, infected mice were anesthetized by inhalation of isoflurane and were intranasally inoculated with 50 µL of ctrl-liposome or clodronate-liposome (0.25 mg/mL) twice with a 3-day interval. Mice were further sacrificed at 4, 15, 31, or 35 d.p.i. for analysis. All animal experiments related to SARS-CoV-2 were performed in a biosafety level 3 laboratory in HKU strictly following SOPs.

### H&E, Masson’s trichrome, and immunofluorescence staining

Tissues collected at necropsy were fixed in zinc formalin and then processed into paraffin-embedded tissue blocks. The tissue sections (4 µm) were stained with H&E and Masson’s trichrome for light microscopy examination. Collagen fibers stain green. The collagen area relative to the wall area was quantified according to the color segmentation by using ImageJ v1.53c as previously described (80). For immunofluorescence staining, tissue sections were first treated with antigen-unmasking solution (Vector Laboratories) in a pressure cooker. After blocking with 0.1% Sudan black B for 15 min and 1% bovine serum albumin (BSA)/PBS at RT for 30 min, the primary antibody, rabbit anti-α-SMA antibody or rabbit anti-mouse CD11b antibody (1:200 dilution with 1% BSA/PBS), was incubated at 4°C overnight. This step was followed by AF568-conjugated goat anti-rabbit IgG for 1 h and then mounted with 4′,6-diamidino-2-phenylindole (DAPI, Thermo Fisher Scientific, Cat# D1306, RRID:AB_2629482). α-SMA (SMA) staining was visualized from fluorescence data using Phenochart Simulated Brightfield mode, rendering SMA-positive cells in a brown IHC-like signal. All tissue sections were examined, the images were captured with a Carl Zeiss LSM780 confocal microscope, and the mean fluorescence intensity (MFI) was further measured by ImageJ v1.53c.

### Real-time qPCR

Lung tissues were homogenized and subjected to the determination of viral load and fibronectin by quantitative real-time PCR (qRT-PCR). Briefly, total RNA was extracted using RNAeasy mini kit (Qiagen) and followed by reverse transcription (PrimeScript II 1st Strand cDNA Synthesis Kit). The real-time RT-PCR assay for SARS-CoV-2-RdRp/Hel RNA, nucleocapsid protein (NP) subgenomic RNA, and fibronectin RNA detection was performed using One-Step TB Green PrimeScript RT-PCR Kit II according to the manufacturer’s instructions. Mouse β-actin was used as normalization.

### Plaque assay

Infectious virus titration was determined by plaque assay. Confluent Vero-E6-TMPRSS2 cells in a 12-well plate were incubated with 10-fold serially diluted tissue homogenates for 1 h. The virus supernatant was discarded, and cells were then overlaid with 1% agarose in DMEM and further incubated for 3 days at 37°C followed by overnight fixation via 4% PFA. Agarose gels were removed, and plaques were visualized by 1% crystal violet.

### Flow cytometry

The cells from lungs of mice were washed one time with staining buffer (PBS contained 2% FBS) followed by staining with three panels of anti-mouse antibodies for 30 min at 4°C. Panel 1: lineage (percp-cy5.5 anti-CD3/CD19/NK1.1/Ly6C), PE-cy7 anti-CD11c, APC anti-CD11b, BV605 anti-B220, BV711 anti-BST2, BV786 anti-CD8, BV421 anti-CD103, APC-Fire750 anti-CD45, PE anti-CD86, APC-Fire750 anti-CD45, BV421 anti-F4/80, PE-cy7 anti-CD11c, APC anti-CD11b, Percp-cy5.5 anti-Ly6C, and BV785 anti-CCR2; Panel 2: APC-Fire750 anti-CD45, BV421 anti-F4/80, PE-cy7 anti-CD11c, APC anti-CD11b, Percp-cy5.5 anti-Ly6C, and BV785 anti-CCR2. Panel 3: APC-Fire750 anti-CD45, PE-cy7 anti-CD3, BV711 anti-CD4, BV786 anti-CD8, percp-cy5.5 anti-NK1.1, AF647 anti-CD19, PE anti-CD69, and BV421 anti-CD103. For bone marrow cell staining, cells were stained with Percp-cy5.5 anti-lineage (CD3/CD19/NK.1.1), BV711 anti-CD117, APC anti-CD135, PE-cy7 anti-CD115, BV786 anti-CD127, BV605 anti-SCA-1, BV421 anti-CD16/32, PE-anti-CD34, and APC-Fire750 anti-CD45. All cells were also stained with dead cell dye (Zombie Aqua, Biolegend Cat# 423102). Stained cells were acquired by FACSAriaIII Flow Cytometer (BD Biosciences) and analyzed with FlowJo software (v10.6) (BD Bioscience).

### Pseudotyped virus-based neutralization assay

To determine the neutralizing activity of mouse sera, specimens were inactivated at 56°C for 30 min before the pseudotyped viral entry assay as previously described (81). In brief, SARS-CoV-2 Omicron BA.1 was generated through co-transfection of 293T cells with two plasmids, pSARS-CoV-2 Spike (Omicron BA.1) and pNL4-3Luc_Env_Vpr, carrying the optimized SARS-CoV-2 S gene and a human immunodeficiency virus type 1 backbone, respectively. At 48 h post-transfection, viral supernatant was collected and frozen at −80°C. Serially diluted serum samples were incubated with 200 TCID50 of pseudovirus at 37°C for 1 h. The serum-virus mixtures were then added to pre-seeded HEK 293T-hACE2 cells. After 48 h, infected cells were lysed, and luciferase activity was measured using Luciferase Assay System kits (Promega) in a Victor3-1420 Multilabel Counter (PerkinElmer). The 50% inhibitory concentrations (IC50) of each specimen were calculated using non-linear regression in GraphPad Prism v8.

### Protein production

GLP1-Fc and ctrl-Fc were provided by Antibody and Immunoassay Services of Li Ka Shing Faculty of Medicine, The University of Hong Kong. Basically, the human GLP1-Fc was created by conjugating human GLP1 (A2G mutation) with human Fc using a (G4S)_3_ linker. The conjugation of human Fc with a (G4S)_3_ linker served as ctrl-Fc. The proteins were confirmed to be endotoxin-free.

### Single-cell RNA-seq data reanalysis

Processed PASC single-cell data with three timepoints, T1 (baseline at clinical diagnosis/hospital admission), T2 (acute disease, around 5 days post-admission), and T3 (convalescent stage, 2–3 months post-symptom onset) were downloaded from the BioStudies database (Accession: E-MTAB-10129). The study cohort consisted of individuals recovering from SARS-CoV-2 infection, stratified into non-T2D (*n* = 79) and T2D (*n* = 11) groups based on clinical history. The annotations for T2D phenotype in the PASC cohort were obtained from the original paper (34). After filtering, 90 PASC samples (non-T2D: *n* = 79; T2D: *n* = 11) with T2D descriptions were preserved for further analysis. Fibrosis-related genes were integrated from previous studies and the FibroAtlas database (82, 83). Analysis procedures were carried out with Seurat v4.3.2 (84). Doublets were removed using scDblFinder (85). To reduce the initial noise, cells with fewer than 200 genes expressed, more than 2,500 genes expressed, more than 10,000 UMI counts identified, or a proportion of mitochondrial gene expression accounting for more than 10% were excluded from the subsequent analysis. Harmony v1.2.0 (86) was used to integrate the samples based on 2,000 of the most variable expression genes across cells. The optimal dimension parameter was discovered using Elbow Plots (the Elbow-Plot function in Seurat). UMAP dimensionality reduction (87) was performed using the top 30 principal components. The Find Clusters function in Seurat was used to obtain cell clusters with the proper resolution. Markers of cells present in the PBMCs were used to determine the appropriate cell types for each cluster. After annotating and combining cell subpopulations, seven distinct cell populations, including monocytes, were identified by clustering. Differentially expressed genes were obtained by the FindMarkers function based on the Wilcoxon rank-sum test. A gene that satisfied the absolute value of log fold-change greater than 0.25, the Bonferroni-adjusted *P*-value less than 0.05, and expressed in more than 10% of the cells, was considered a differentially expressed gene. Enriched GO pathways were assessed using the R package clusterProfiler v4.10.0 (88).

### PASC proteomics data reanalysis

The proteomics data for the PASC cohort were obtained from the original paper (34). T-test was used to evaluate the significance between the non-T2D and T2D groups across the three timepoints.

### Preparation and culture of BMDM from the mouse

BMDM was prepared and cultured according to the protocol as previously described (89). Briefly, mice were euthanized by injection of pentobarbital sodium. Bone marrows were washed out by 2 mL of PBS from the end of the femur and tibia with a 23G needle after removal of epiphyses. Erythrocytes were removed by 1 × lyse buffer on ice. Bone marrows were then washed with PBS and seeded in 12-well plates (3–5 × 10^5^ cell/well) with DMEM medium (10% FBS, 1% penicillin/streptomycin, and 1 ng/mL of M-CSF1). Half volume of medium was added to the cells on day 4. Bone marrow-derived macrophages were ready for use at day 7.

### RNA-seq analysis

BMDMs were exposed with MA-BA.1 (MOI:1) for 24 h in the presence or absence of GLP1-Fc treatment (50 ng/mL). Total RNA of BMDMs was extracted by RNeasy Mini Kit (QIAGEN) according to the instructions. Sample QC, library preparation, and Illumina sequencing (pair-end sequencing of 151 bp) were done at Centre for PanorOmic Sciences (CPOS), Genomics Core, LKS Faculty of Medicine, The University of Hong Kong. Raw fastq data were quality-controlled using FastQC v0·11·7 (http://www.bioinformatics.babraham.ac.uk/projects/fastqc/) and fastp (90). The clean reads were aligned to the UCSC GRCm38 reference using Hisat2 v2.2.1 (91). HTSeq v0.6.1 was used to generate raw read counts for each gene (92). The differential expression analysis was carried out using DESeq2 (93). Genes with |log2 fold change| > 0.263 and adjusted *P*-value < 0.05 (FDR) were identified as significantly different. GSEA was performed with the fgsea R package (https://github.com/ctlab/fgsea).

### Statistical analysis

Statistical analysis was performed with the GraphPad Prism 8 Software. Data are represented by mean values or mean values with SEM. Significant differences between the two groups were determined using the two-tailed Student’s t-test. *P* < 0.05 was considered statistically significant. Correlation analysis was performed by linear regression using GraphPad Prism 8.0.

## Data Availability

The RNA sequencing data have been deposited in the National Center for Biotechnology Information Gene Expression Omnibus under accession code GSE286940. All other raw data are available upon request from the corresponding authors.

## SUPPLEMENTAL MATERIAL

Supplemental material jvi.00401-26-s0001.pdf Fig. S1 to S7.

ASM does not own the copyrights to Supplemental Material that may be linked to, or accessed through, an article. The authors have granted ASM a non-exclusive, world-wide license to publish the Supplemental Material files. Please contact the corresponding author directly for reuse.
