# Supplementary material for: Glucagon-like peptide-1 receptor agonist prevents pulmonary fibrosis following acute COVID-19 infection associated with type 2 diabetes
Source: J Virol. 2026 Jul 9;100(8):e00401-26. doi: 10.1128/jvi.00401-26 (PMC13483299; doi:10.1128/jvi.00401-26)
Supplement: Supplementary file 1 [file jvi.00401-26-s0001.pdf]

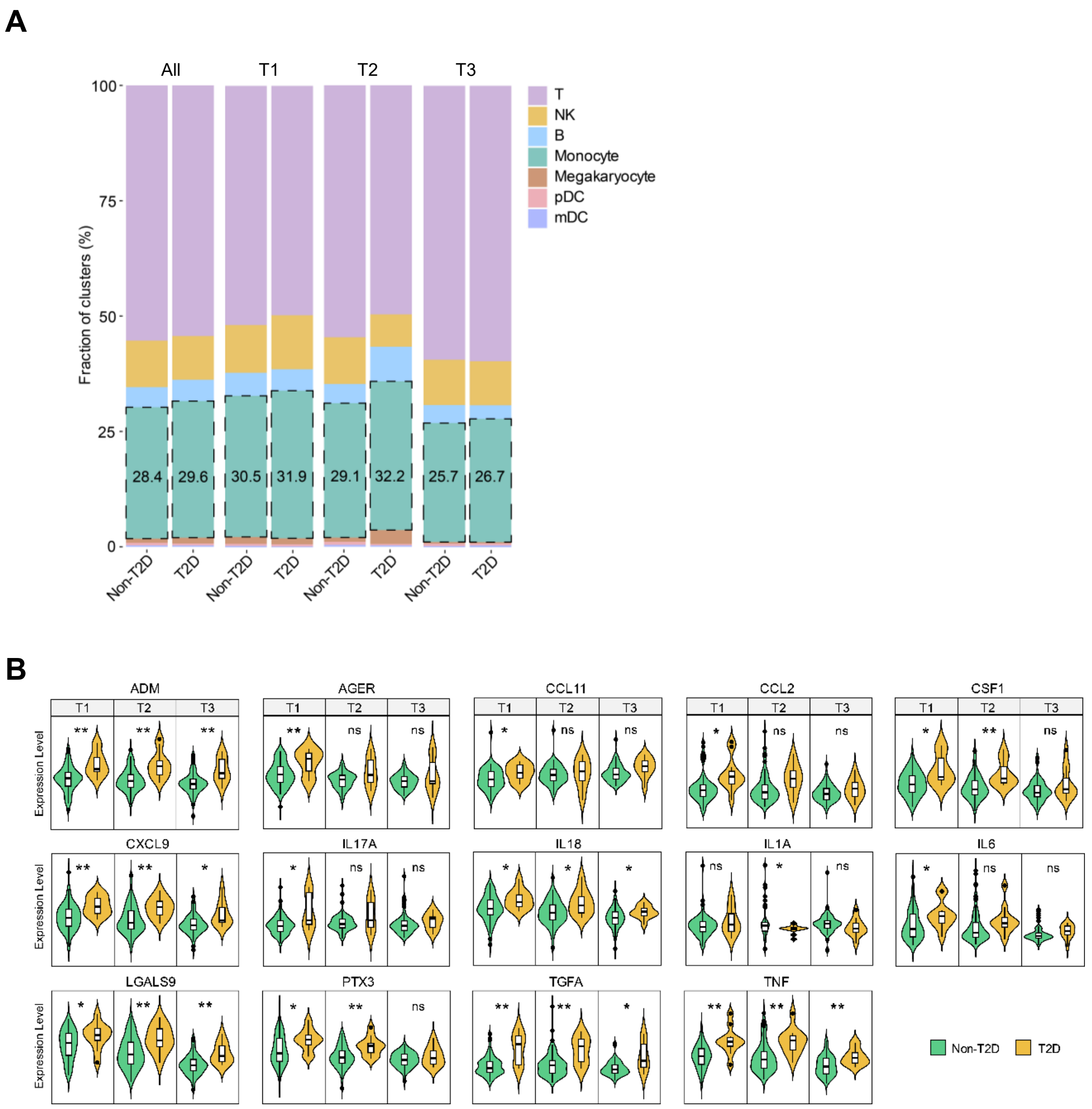

Fig. S1. Comparison of immune cells and plasma cytokines between T2D and non-T2D individuals with PASC. (**A**) Frequencies of different immune cells from PBMCs between non-T2D and T2D individuals across three timepoints. (**B**) Plasma cytokine expression between non-T2D and T2D individuals across three timepoints. *P*-values calculated from the *t* test are displayed. ns p>0.05, *p<0.05, **p< 0.01, ***p< 0.001.

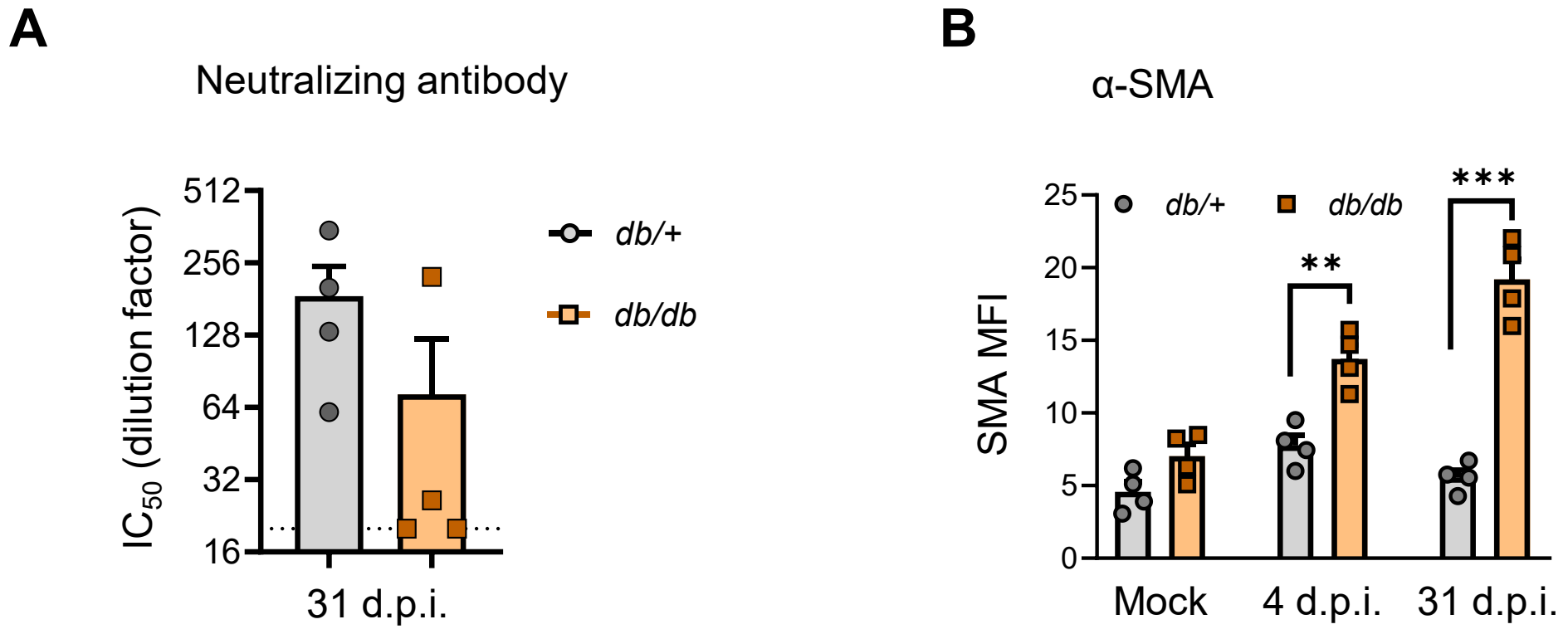

Fig. S2. The neutralizing antibody titer in blood and α-smooth muscle actin (α-SMA) expression in lungs from db/+ and db/db mice.

(A) Blood samples were collected from mice at 31 d.p.i. and subjected to measurement of neutralizing activity against pseudotyped Omicron BA.1; the dashed line indicates the detection limit.

(B) The extent of α-smooth muscle actin (α-SMA) staining in the lung was quantified as mean fluorescent intensity (MFI) in mock-infected mice and in mice infected with SARS-CoV-2 at 4 and 31 days post infection (d.p.i.).

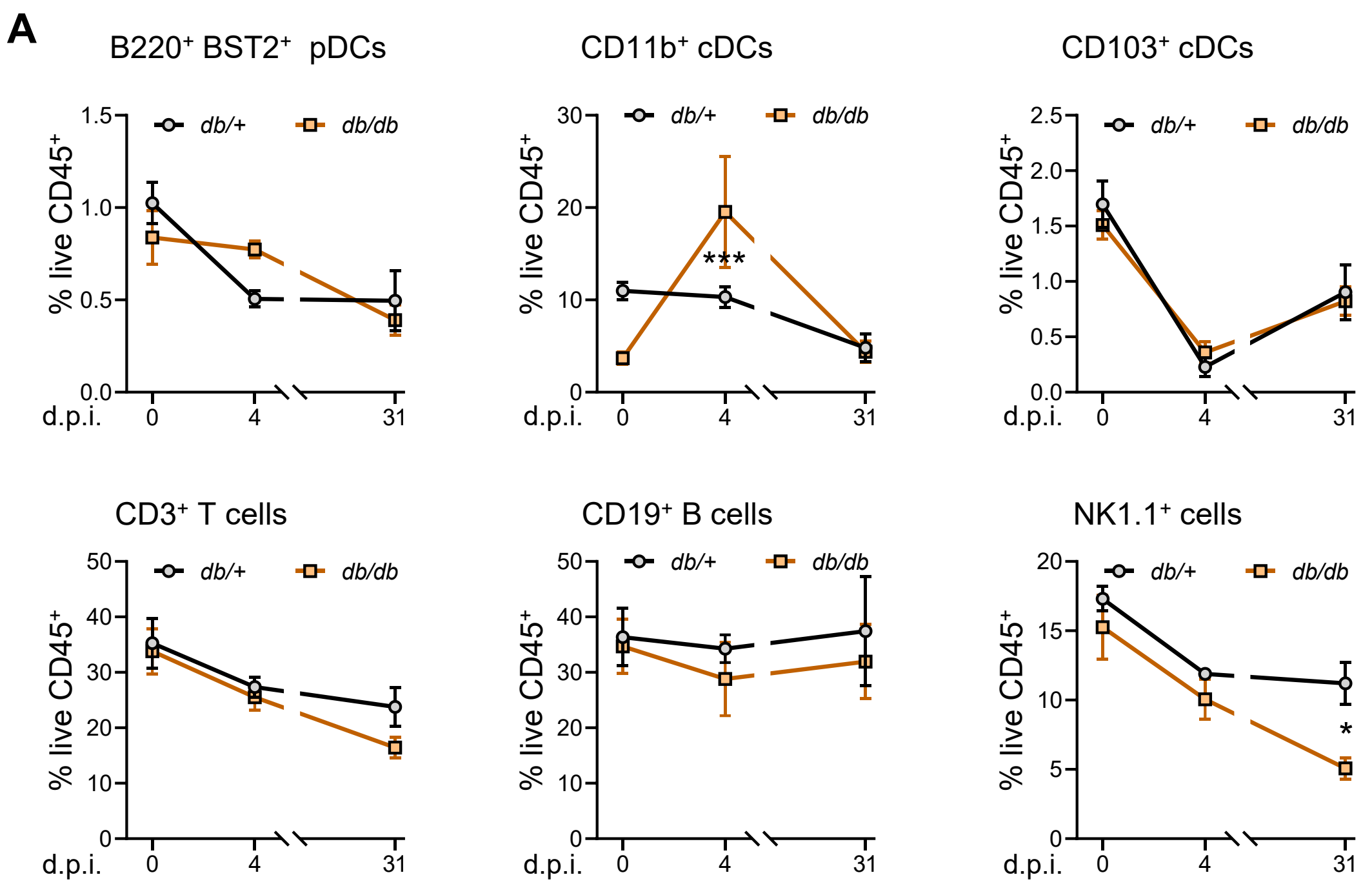

Fig. S3. Comparison of immune cells in lungs between db/+ and db/db mice after SARS-CoV-2 infection. (**A**) Frequencies of B220⁺BST2⁺ pDCs, CD11b⁺ cDCs, CD103⁺ cDCs, CD3⁺ T cells, CD19⁺ B cells and NK1.1⁺ NK cells were gated on CD45⁺ live cells in db/+ and db/db mice at 0, 4 and 31 d.p.i. *P*-values calculated by the *t* test are displayed. **p*<0.05, ***p*< 0.01.

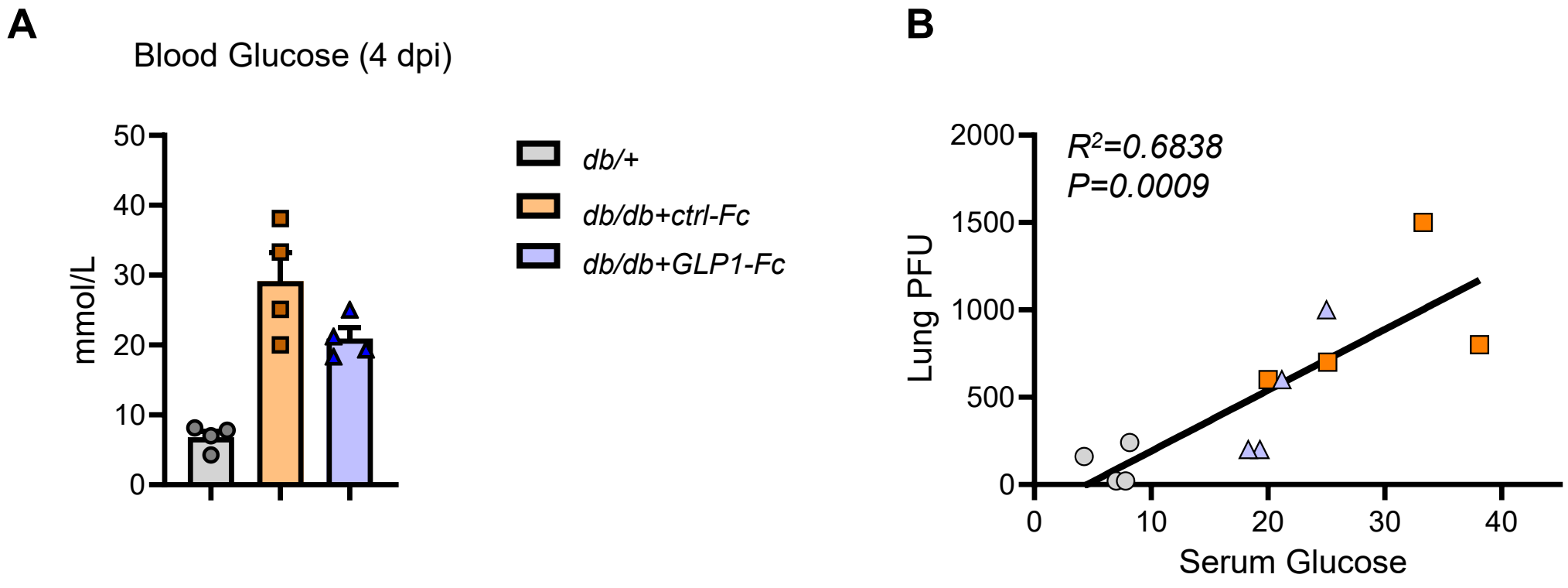

Fig. S4. Blood glucose of db/+ and db/db mice with ctrl-Fc or GLP1-Fc at 4.p.pi. (A) Mouse serum was collected at 4 d.p.i. and subjected to glucose measurement. (**B**) Correlate analysis between serum glucose and live virus PFU was performed by linear regression using GraphPad Prism 8.0. Each symbol represents an individual mouse with color-coding for corresponding groups.

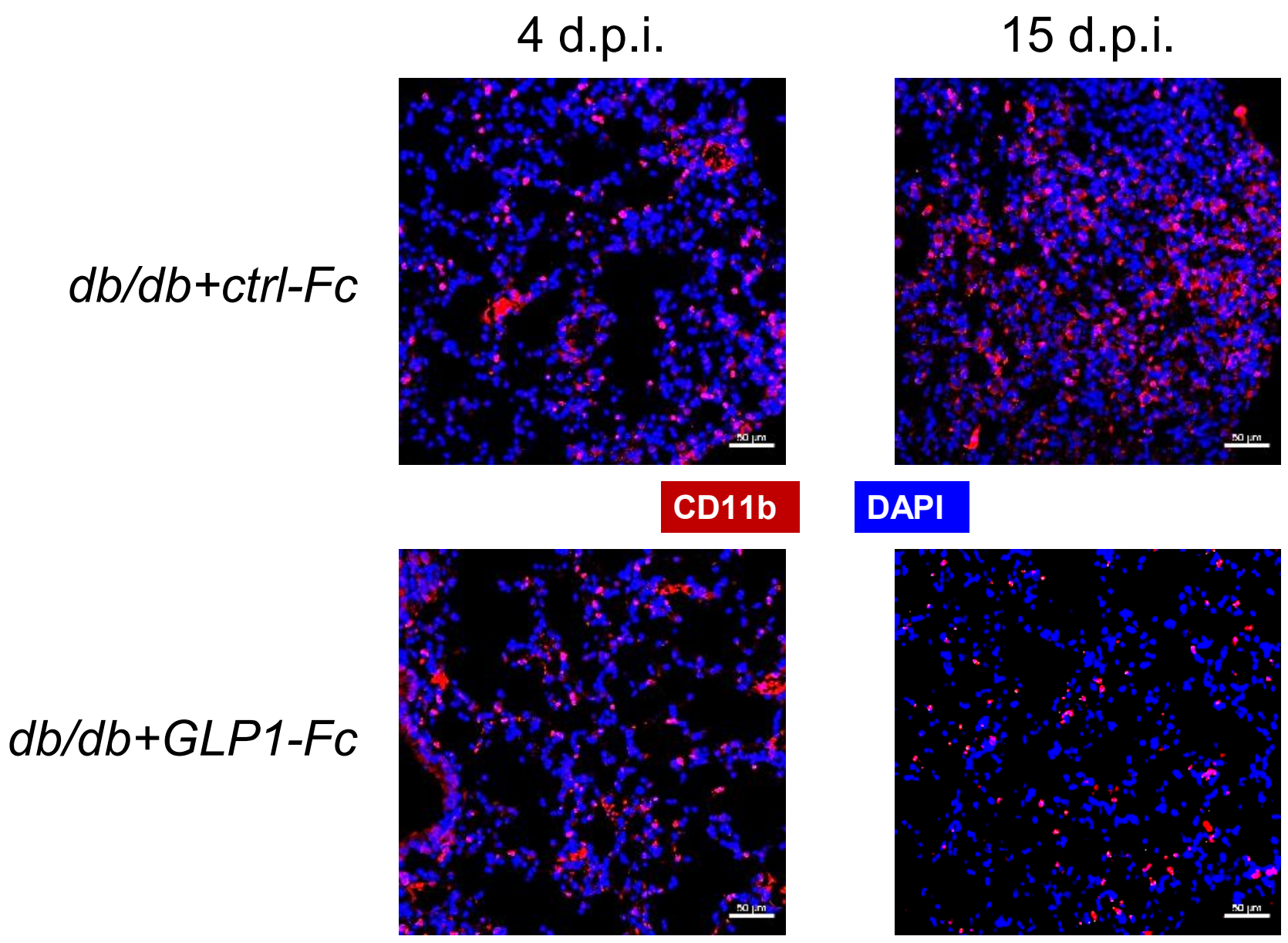

Fig. S5. Confocal images showed CD11b^{+} (red) cells in lungs from db/db mice that received ctrl-Fc or GLP1-Fc at 4 and 15 d.p.i. Scale bars: 50 μm.

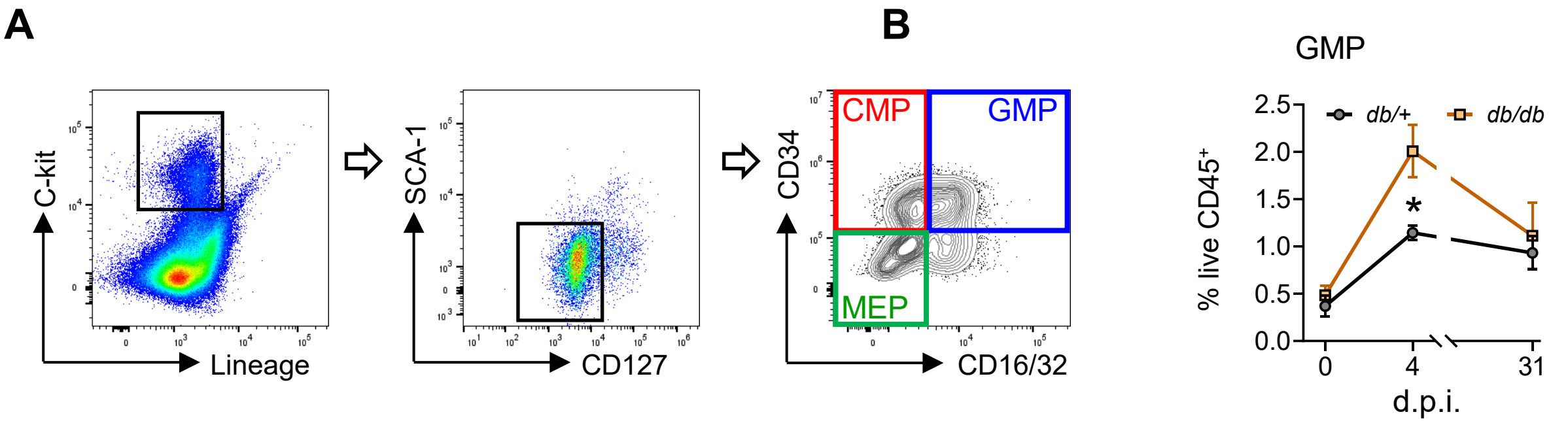

Fig. S6. Frequencies of granulocyte-monocyte progenitors (GMPs) in bone marrow between db/+ and db/db mice after SARS-CoV-2 infection. (**A**) Bone marrow cells were isolated and subjected to flow cytometry staining. Representative plots show the gating strategy of common myeloid progenitor (CMP), granulocyte-monocyte progenitor (GMP) and Megakaryocyte–erythroid progenitor (MEP). (**B**) Frequencies of GMP were gated on live $CD45^+$ cells in db/+ and db/db mice at 0, 4 and 31 d.p.i. Statistics were generated using *t* test. $*p<0.05$.

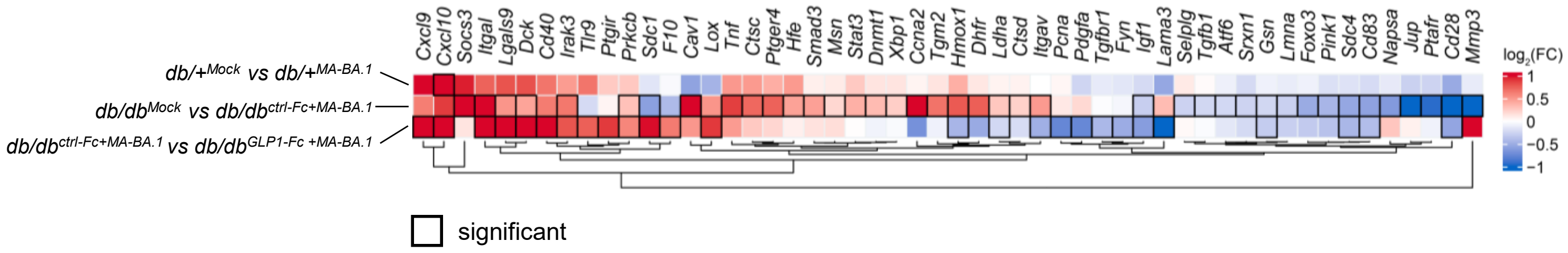

Fig. S7. Differential expressed fibrosis-associated genes among comparisons. Black borders represent significant differences in comparisons. Red and blue intensities represent downregulation and upregulation of genes, respectively.
